# Cognitive function trajectories and influencing factors in Chinese older adults with self-reported hearing impairment: findings from CHARLS 2013–2020

**DOI:** 10.1186/s12877-025-06795-8

**Published:** 2025-11-29

**Authors:** Xuan Huang, Cui Ye, Ziyu Wang, Shufang Zuo, Yong Xu, Jinghong Liang, Martine Puts, Lu Lin

**Affiliations:** 1 The First Affiliated Hospital of Soochow University, No. 188 Shizi Street, Suzhou, 215006 China; 2 School of Nursing, Suzhou Medical College of Soochow University, Suzhou, China; 3 School of Public Health, Suzhou Medical College of Soochow University, Suzhou, China; 4 School of Public Health, Peking University, Beijing, China; 5 Lawrence S. Bloomberg Faculty of Nursing, University of Toronto, Toronto, Canada

**Keywords:** Hearing impairment, Cognitive trajectory, Influencing factor, Longitudinal analysis, Group-based trajectory modeling

## Abstract

**Objectives:**

Hearing impairment is linked to an increased risk of cognitive decline, yet the progression of cognitive function in affected individuals remains unclear. This study examined cognitive function trajectories in older adults with self-reported hearing impairment and identified key influencing factors.

**Methods:**

Data from China Health and Retirement Longitudinal Study (CHARLS, 2013–2020) were analyzed. Sociodemographic and health-related factors, including age, education, sensory impairments, chronic conditions, hearing aid use, depression, vision, sleep patterns, social interactions, smoking, drinking, retirement status, and fuel use, were assessed. Cognitive function was evaluated using episodic memory, orientation, attention, and executive function. Group-Based Trajectory Modeling (GBTM) identified cognitive trajectories, while logistic regression examined influencing factors.

**Results:**

Three cognitive trajectories were identified: low-functioning decline (L, 23.5%), middle-functioning decline (M, 38.5%), and high-functioning stabilization (H, 38.0%). The likelihood of M increased with younger age (60–69 years: OR = 8.56; 70–79 years: OR = 4.54), absence of physical (OR = 2.03) or visual impairment (OR = 1.65), and short naps (≤ 30 min, OR = 1.59). H was associated with younger age (60–69 years: OR = 60.36; 70–79 years: OR = 12.11), absence of physical (OR = 2.20) or visual disability (OR = 1.84), mild depression (OR = 3.91), and shorter naps (OR = 2.05). Poor hearing (OR = 0.46), illiteracy (OR = 0.03), and non-retirement (OR = 0.23) reduced the likelihood of being in the M or H groups (all *p* < 0.05).

**Conclusions:**

Cognitive trajectories in hearing-impaired older Chinese adults fall into three categories: low-functioning decline, middle-functioning decline, and high-functioning stabilization. Age, education, sensory impairments, depression, nap duration, and retirement status influence these trajectories. Given the limited generalizability of these preliminary findings, further research is needed to clarify the potential confounding and mediating relationships among these factors before they can inform early intervention and policy initiatives.

## Introduction

As individuals age, cognitive function tends to decline to varying degrees, particularly in areas such as processing speed, executive function, and both episodic and working memory. Accelerated cognitive decline is closely associated with an increased risk of dementia in older adults and may reflect common neurodegenerative changes or other age-related factors [1, 2]. It is estimated that the number of dementia patients in China will reach approximately 36 million by 2050 [3]. Evidence suggests that hearing impairment is an independent and potentially modifiable risk factor for dementia [4, 5]. It is reported that approximately 430 million people worldwide suffer from hearing impairment, and by 2050, this number is projected to exceed 700 million, representing one-tenth of the global population [6]. Studies indicate that the pooled prevalence of hearing impairment among middle-aged and older adults in China is 45%, accounting for nearly half of this population [7], with approximately 64.9% of older adults having hearing levels below normal or average [8].

In older adults with hearing impairment, long-term auditory deprivation may potentially reduce the white matter microstructural integrity in brain regions critical for cognitive processing, leading to declines in global cognitive functioning and specific cognitive domains such as executive function, processing speed, memory, and episodic memory [5, 9–12]. This is further supported by a meta-analysis of the Chinese population, which found that self-reported hearing impairment was negatively correlated with cognitive performance [13]. The prevalence of cognitive impairment is 4.2% higher in older adults with hearing impairment compared to those with normal hearing [14]. Additionally, the relationship between hearing and cognitive function may be mediated by other potential dementia risk factors, including education, social isolation, and depression [15].

Currently, several theories have been proposed to explain the association between hearing impairment and cognitive decline [16]. The common cause hypothesis suggests that age-related deterioration of sensory organs and neural functions leads to concurrent declines in both hearing and cognitive abilities [17]. The cognitive load hypothesis suggests that hearing impairment consumes additional cognitive resources to compensate for hearing deficits, which crowds out resources needed for higher-level cognitive tasks such as memory [18, 19]. The sensory deprivation hypothesis emphasizes that prolonged auditory input reduction results in insufficient stimulation of key cognitive centers (e.g., the frontal and temporal lobes), ultimately leading to functional decline [19, 20]. In addition, the social isolation hypothesis [21] suggests that hearing loss may indirectly affect cognitive function by reducing social interaction and increasing feelings of loneliness and the risk of depression. These theoretical frameworks provide potential mechanistic perspectives for this study to explore the heterogeneity of cognitive trajectories among older adults with hearing impairment and its influencing factors.

We hypothesized that cognitive function trajectories in older adults with self-reported hearing impairment exhibit heterogeneous patterns and are significantly influenced by multiple factors, including demographic characteristics, health behaviors, and psychosocial variables. Accurate identification of these trajectories and their determinants could provide critical evidence for developing targeted intervention strategies, thereby helping to slow cognitive decline in this population.

## Methods

### Design and participants

CHARLS is a large-scale interdisciplinary survey project jointly conducted by Wuhan University and Peking University in China, which is widely recognized as one of the most authoritative data sources for research on China’s aging population [22]. It uses a stratified multistage probability-proportional random sampling strategy to survey middle-aged and older adults aged 45 and older across 28 provinces, autonomous regions, and municipalities nationwide. The sampling strategy ensures that the data are highly representative of China’s middle-aged and older population in terms of gender, age, urban-rural distribution, education, and socioeconomic status. The baseline survey was conducted in 2011, with follow-up surveys every 2–3 years. Data collection for CHARLS was conducted by university students who completed 60 h of systematic training and passed qualification exams. This training ensured proficiency in questionnaire management systems, comprehensive knowledge of survey content, professional interviewing skills, and mastery of field investigation procedures. To further enhance data quality, CHARLS prioritized recruiting interviewers with local dialect proficiency and assigned them to conduct face-to-face interviews in their hometowns or neighboring regions, thereby reducing language barriers. Face-to-face interviews were used to ensure both the accuracy and depth of the collected information. According to official CHARLS reports, the overall baseline response rate was 80.5%, including 94% in rural areas and 69% in urban areas. The CHARLS survey was approved by the Biomedical Ethics Committee of Peking University (approval number: IRB00001052-11015), and all participants provided written informed consent.

**Fig. 1:**
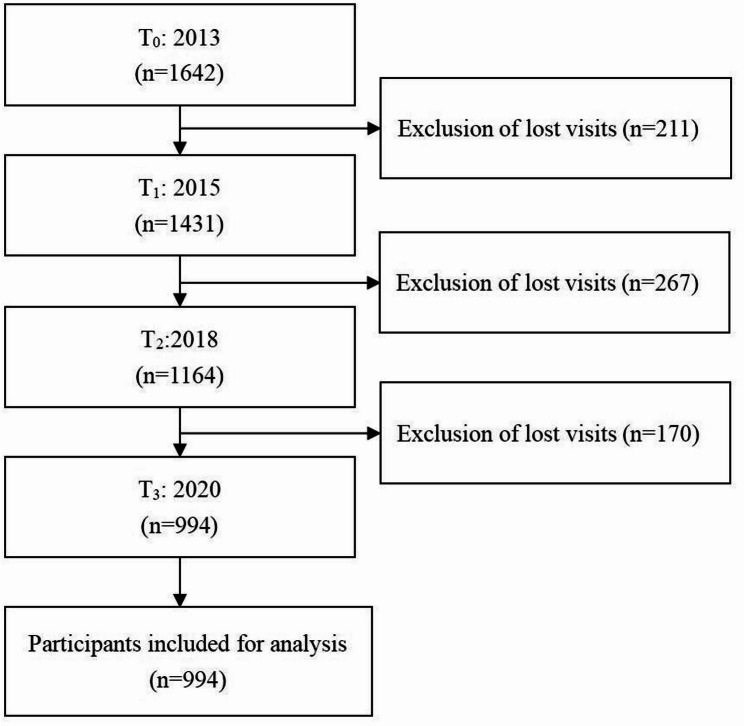
Flowchart for screening study participants

This study used data from the CHARLS database across four waves: 2013 (T_0_), 2015 (T_1_), 2018 (T_2_), and 2020 (T_3_). Participants were included if they met the following criteria: (1) aged ≥ 60 years with self-reported hearing impairment at baseline in 2013; (2) complete cognitive function data available at all four time points (2013, 2015, 2018, and 2020). In the 2013 baseline survey, a total of 1,642 participants met the inclusion criteria. At baseline, 1,642 participants met the inclusion criteria; 1,431, 1,164, and 994 completed cognitive assessments in 2015, 2018, and 2020, respectively. Ultimately, 994 participants were included in the cognitive function trajectory analysis (see Fig. 1).

### Data collection

#### Cognitive function

The cognitive function scores of hearing-impaired older adults from the CHARLS database in 2013, 2015, 2018, and 2020 were selected as the dependent variable for this study. Cognitive function was assessed using the Telephone Interview for Cognitive Status (TICS) and administered through face-to-face interviews by investigators who had completed standardized training [22], comprising episodic memory (10 points) and a composite measure of orientation (5 points), attention (5 points), and executive function (1 point). For the episodic memory test, participants were asked to recall 10 unrelated words, with scores ranging from 0 to 10. The orientation test was based on participants’ responses to questions about the current year, month, date, day of the week, and season, with a score range of 0 to 5. The attention test was conducted by asking participants to subtract 7 from 100 up to five times, awarding one point for each correct response, with a total score range of 0 to 5. For the executive function test, participants were asked to replicate a given shape, receiving one point for a correct reproduction. The total cognitive function score was the sum of the above-mentioned scores, with a maximum score of 21 points, where higher scores indicated better cognitive function. This method was consistently used to assess cognitive function at each follow-up year.

#### Sociodemographic and health-related variables

Twenty-seven sociodemographic and health-related variables were extracted from the CHARLS database for the year 2013 (baseline), including demographic variables (age, gender, education, residence, marital status), various impairments (physical, intellectual, visual), duration of impairment, chronic comorbidities, hearing aid use, self-assessed memory and hearing, depression, life satisfaction, vision (farsightedness, nearsightedness), complete tooth loss, history of falls, sleep patterns (night sleep, nap duration), social interactions, smoking, drinking, frequency of contact with children, retirement status, and fuel use.

### Data analysis

The Group-Based Trajectory Model (GBTM) is a latent class growth model used to analyze longitudinal data and explore heterogeneity [23]. Its underlying principle assumes population heterogeneity, meaning that within a population, there are several latent subgroups with distinct developmental trajectories or patterns. The goal is to identify how many subgroups exhibit different developmental trends. The number of subgroups, treated as a latent categorical variable, is estimated by the model to provide the best fit. In this study, the Traj package in Stata software was used for modeling, starting with lower-order models and gradually fitting to determine the optimal number of trajectory groups and identify the structure of each group [24]. Selecting the best model requires a combination of factors [25]: (a) Statistical significance (*P* < 0.05 for model parameters); (b) Bayesian Information Criterion (BIC), where smaller absolute values indicate better model fit [25, 26]; (c) Average Posterior Probability (AvePP), which reflects the mean posterior probability of each individual being assigned to a particular group. An AvePP >0.7 indicates acceptable model accuracy [27]; (d) The sample proportion of each subgroup should generally be not less than 5%; (e) Visual inspection of the predicted trajectories (in conjunction with clinical relevance); (f) Entropy greater than 0.8, which reflects good separation between subgroups [28].

The sociodemographic and health-related factors of the participants were selected as independent variables, which were categorical variables presented as frequencies and percentages. Univariate analyses used the χ^2^ test or the Kruskal-Wallis (K-W) test to compare distributional differences between trajectory groups. For regression analysis, the types of cognitive function change trajectories were used as the dependent variable, while the statistically significant variables from univariate analyses (those with significant baseline distribution differences between trajectory groups) served as independent variables. Multivariable logistic regression was used to examine the relationship between the dependent and independent variables. Two-sided tests were applied, and results were considered statistically significant at *P* < 0.05.

## Results

### Sociodemographic characteristics of participants

This study included 994 older adults with self-reported hearing impairment, consisting of 509 males and 485 females, with a mean age of 69.45 ± 7.18 years. Among them, 771 (77.57%) were married, and 472 (47.48%) were illiterate. The mean baseline cognitive function score was 7.46 ± 5.16. The sociodemographic characteristics of all the participants, as well as a comparison of the baseline characteristics of participants across different cognitive function change trajectories, are presented in Table 1. Statistically significant differences (*P* < 0.05) were observed between groups for all variables, except for intellectual impairment, number of chronic comorbidities, hearing aid use, life satisfaction, falls, and frequency of contact with children.

**Table 1:** Sociodemographic characteristics of older adults with self-reported hearing impairment across different trajectories of cognitive function changes

Variable	Classification	N(%)	Trajectory group [n(%)]			X^2^/H	P
			Low-start decline (*n* = 232)	Middle-start decline (*n* = 387)	High-start stabilization (*n* = 375)		
Age (years)	60–69	564(56.7)	73(31.5)	212(54.8)	279(74.4)	138.988	< 0.001
	70–79	321(32.3)	88(37.9)	147(38.0)	86(22.9)		
	≧ 80	109(11.0)	71(30.6)	28(7.2)	10(2.7)		
Gender	Female	485(48.8)	152(65.5)	208(53.8)	125(33.3)	65.643	< 0.001
	Male	509(51.2)	80(34.5)	179(46.2)	250(66.7)		
Education	Illiterate	472(47.5)	187(80.6)	236(61.0)	49(13.1)	333.232	< 0.001
	Primary school and below	396(39.8)	40(17.2)	135(34.9)	221(58.9)		
	Junior high school	83(8.4)	2(0.9)	12(3.1)	69(18.4)		
	Senior High school and above	43(4.3)	3(1.3)	4(1.0)	36(9.6)		
Residence	Rural	870(87.5)	215(92.7)	354(91.5)	301(80.3)	29.248	< 0.001
	Urban	124(12.5)	17(7.3)	33(8.5)	74(19.7)		
Marital status	Unmarried (Single/Divorced/Widowed)	223(22.4)	87(37.5)	81(20.9)	55(14.7)	43.766	< 0.001
	Married	771(77.6)	145(62.5)	306(79.1)	320(85.3)		
Physical impairment	No	893(89.8)	193(83.2)	356(92.0)	344(91.7)	14.672	0.001
	Yes	101(10.2)	39(16.8)	31(8.0)	31(8.3)		
Intellectual impairment	No	883(88.8)	202(87.1)	346(89.4)	335(89.3)	0.95	0.622
	Yes	111(11.2)	30(12.9)	41(10.6)	40(10.7)		
Visual impairment	No	745(75.0)	160(69.0)	290(74.9)	295(78.7)	7.185	0.028
	Yes	249(25.0)	72(31.0)	97(25.1)	80(21.3)		
Duration of impairment (years)	1–5	459(46.2)	93(40.1)	197(50.9)	169(45.1)	7.122	0.028
	> 5	535(53.8)	139(59.9)	190(49.1)	206(54.9)		
Number of chronic co-morbidities	0–1	351(35.3)	94(40.5)	135(34.9)	122(32.5)	4.050	0.133
	≥ 2	643(64.7)	138(59.5)	252(65.1)	253(67.5)		
Hearing aid use	No	948(95.4)	221(95.3)	368(95.1)	359(95.7)	0.187	0.911
	Yes	46(4.6)	11(4.7)	19(4.9)	16(4.3)		
Self-assessed memory	Poor	488(49.1)	125(53.8)	222(57.4)	141(37.6)	24.233	< 0.001
	Fair	396(39.8)	79(34.1)	125(32.3)	192(51.2)		
	Good	110(11.1)	28(12.1)	40(10.3)	42(11.2)		
Self-assessed hearing*	Poor	575(57.8)	166(71.6)	224(57.9)	185(49.3)	26.595	< 0.001
	Fair	334(33.6)	53(22.8)	125(32.3)	156(41.6)		
	Good	85(8.6)	13(5.6)	38(9.8)	34(9.1)		
Depression score	0–10	669(67.3)	161(69.4)	238(61.5)	270(72.0)	11.204	0.004
	11–20	288(29.0)	60(25.9)	129(33.3)	99(26.4)		
	21–30	37(3.7)	11(4.7)	20(5.2)	6(1.6)		
Life satisfaction	Dissatisfied	157(15.8)	39(16.8)	78(20.2)	40(10.7)	2.833	0.243
	Partially satisfied	551(55.4)	116(50.0)	199(51.4)	236(62.9)		
	Satisfied	286(28.8)	77(33.2)	110(28.4)	99(26.4)		
Myopia	Poor	380(38.2)	95(41.0)	168(43.4)	117(31.2)	14.41	0.001
	Fair	532(53.5)	126(54.3)	188(48.6)	218(58.1)		
	Good	82(8.3)	11(4.7)	31(8.0)	40(10.7)		
Farsightedness	Poor	279(28.1)	68(29.3)	118(30.5)	93(24.8)	6.167	0.046
	Fair	628(63.2)	147(63.4)	242(62.5)	239(63.7)		
	Good	87(8.7)	17(7.3)	27(7.0)	43(11.5)		
Complete tooth loss	No	760(76.5)	156(67.2)	293(75.7)	311(82.9)	19.785	< 0.001
	Yes	234(23.5)	76(32.8)	94(24.3)	64(17.1)		
History of falls	No	780(78.5)	176(75.9)	303(78.3)	301(80.3)	1.658	0.437
	Yes	214(21.5)	56(24.1)	84(21.7)	74(19.7)		
Night sleep (h)	≦ 7	773(77.8)	54(23.3)	147(38.0)	135(36.0)	7.591	0.022
	> 7	221(22.2)	126(54.3)	143(37.0)	168(44.8)		
Nap duration (min)	≦ 30	506(50.9)	95(41.0)	208(53.8)	203(54.1)	12.005	0.002
	> 30	488(49.1)	137(59.0)	179(46.2)	172(45.9)		
Social interactions	No	502(50.5)	130(56.0)	202(52.2)	170(45.3)	7.293	0.026
	Yes	492(49.5)	102(44.0)	185(47.8)	205(54.7)		
Smoking	Never	520(52.3)	151(65.1)	211(54.5)	158(42.2)	31.541	< 0.001
	Former smoker	212(21.3)	36(15.5)	78(20.2)	98(26.1)		
	Current smoker	262(26.4)	45(19.4)	98(25.3)	119(31.7)		
Drinking	Never	546(54.9)	150(64.7)	233(60.2)	163(43.5)	35.733	< 0.001
	Former drinker	134(13.5)	26(11.2)	52(13.4)	56(14.9)		
	Current drinker	314(31.6)	56(24.1)	102(26.4)	156(41.6)		
Frequency of contact with children	Occasionally	250(25.2)	50(21.6)	103(26.6)	97(25.9)	2.181	0.336
	Often	356(35.8)	84(36.2)	136(35.2)	136(36.2)		
	Always	388(39.0)	98(42.2)	148(38.2)	142(37.9)		
Retirement	No	872(87.7)	223(96.1)	360(93.0)	289(77.1)	64.842	< 0.001
	Yes	122(12.3)	9(3.9)	27(7.0)	86(22.9)		
Fuel use	Clean fuels	220(22.1)	37(16.0)	77(19.9)	106(28.3)	14.458	< 0.001
	Solid fuels	774(77.9)	195(84.0)	310(80.1)	269(71.7)		

*“Self-assessed hearing” refers to the participant’s evaluation of their hearing ability while using a hearing aid (if they typically use one) or without a hearing aid (if they do not)

### Modeling of cognitive function change trajectories

**Fig. 2:**
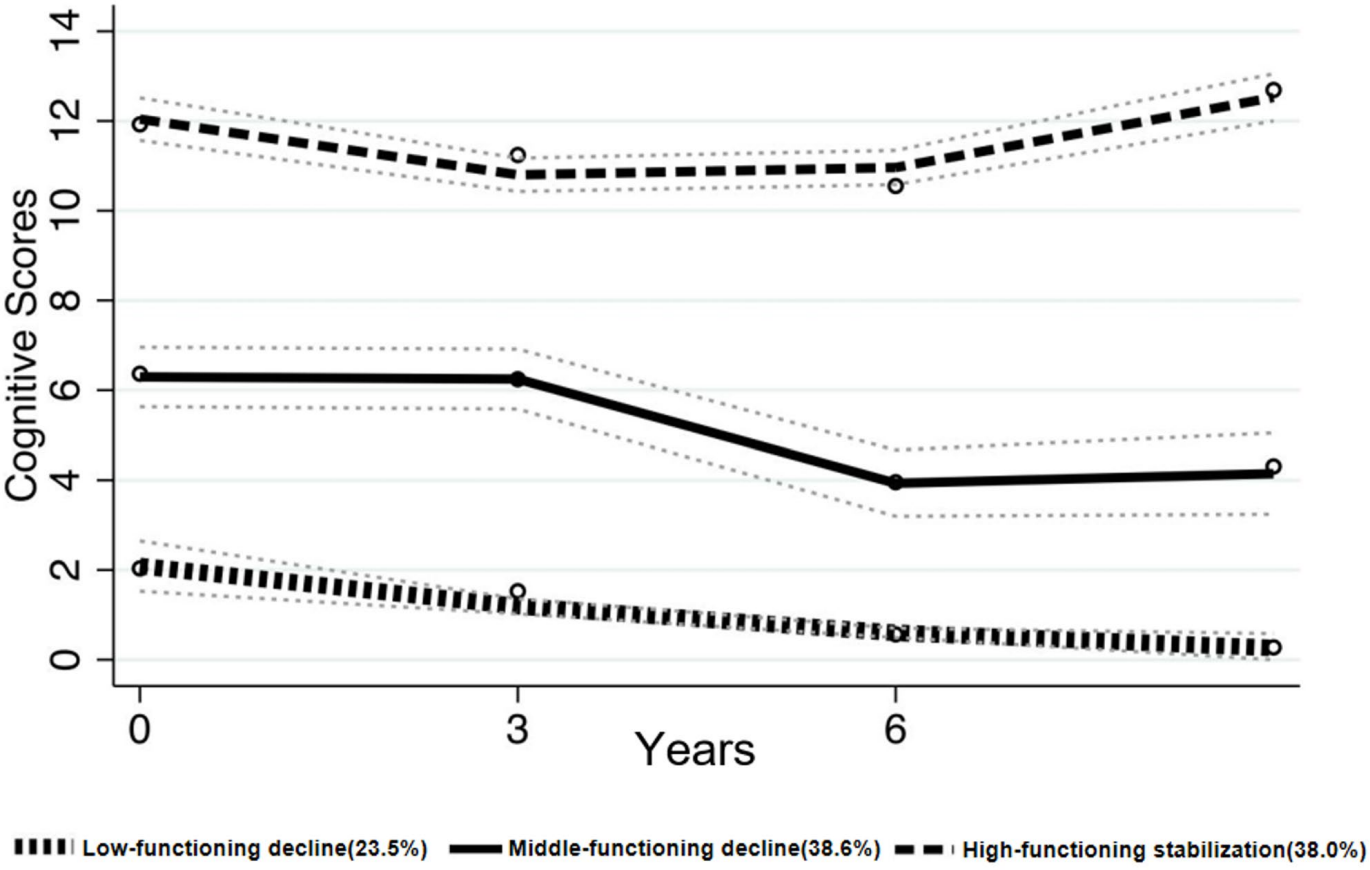
Trajectories of cognitive function changes in older adults with self-reported hearing impairment

Based on the model fit indices presented in Table 2, a three-class model was selected to characterize trajectories of cognitive function changes. While the one-class model has the lowest AIC, BIC, and aBIC values, it assumes homogeneity and does not capture potential heterogeneity in cognitive trajectories. The two-class model demonstrates good separation, with an average posterior probability (AvePP) of 0.97 and entropy of 0.88, but may oversimplify the underlying patterns. The three-class model balances model fit and interpretability, with acceptable fit indices (AIC= -10041.79, BIC=-10071.2, aBIC=-10079.52), reasonably high AvePP (0.92), and entropy (0.824), while also identifying three distinct and meaningful trajectory groups that reflect heterogeneity in cognitive function changes over time. Therefore, the three-class model provides a more nuanced and clinically informative classification of cognitive trajectories. Cognitive function changes over time in older adults with self-reported hearing impairment followed three distinct trajectories (Fig. 2): the Low-functioning decline group (L group) (*n* = 232, 23.5%), the Middle-functioning decline group (M group) (*n* = 387, 38.6%), and the High-functioning stabilization group (H group) (*n* = 375, 38.0%).

**Table 2:** Parameters of the fitted model for different trajectories of cognitive function changes

Number of categories	AIC	BIC	aBIC	AvePP	Entropy	Composition by category [*n* (%)]		
						1	2	3
1-class	-11154.32	-11164.13	-11166.9	1	-	3976(100)	-	-
2-class	-10240.7	-10262.76	-10268.99	0.97	0.88	1960(49.3)	2016(50.7)	-
3-class	-10041.79	-10071.2	-10079.52	0.92	0.824	934(23.5)	1535(38.6)	1510(38.0)

### Multiple logistic regression analysis of factors influencing trajectories of cognitive function changes

No multicollinearity was found among the variables that were statistically significant in the univariate analysis. Consequently, multivariable logistic regression was performed. Using the L group as the reference, the analysis identified several key factors influencing cognitive function changes in hearing-impaired older adults. The detailed results are presented in Table 3. The findings suggest that younger age (60–69 years), middle age (70–79 years), absence of physical and visual impairments, lower depression scores (1–10 and 11–20), and shorter nap durations (≤ 30 min) served as protective factors for cognitive function in this population. Conversely, illiteracy, poor hearing, fair farsightedness, and non-retirement were identified as risk factors.

**Table 3:** Factors influencing the cognitive function change trajectories in Self-Reported older adults with hearing impairment

Middle-functioning decline group					High-functioning stabilization group			
Variable	β	*P*	OR	95% CI	β	*P*	OR	95% CI
Age								
60–69	2.14	< 0.001	8.56	4.56,16.05	4.10	< 0.001	60.36	22.2, 164.02
70–79	1.51	< 0.001	4.54	2.52,8.18	2.49	< 0.001	12.11	4.58, 32.00
≧ 80			1.00				1.00	
Gender								
Female	-0.20	0.475	0.82	0.47,1.42	-0.19	0.579	0.83	0.42,1.62
Male			1.00				1.00	
Education								
Illiterate	-0.16	0.860	0.85	0.14,5.25	-3.44	< 0.001	0.03	0.01, 0.18
Primary school and below	0.82	0.382	2.26	0.36,14.14	-0.37	0.671	0.69	0.13, 3.77
Junior high school	1.05	0.377	2.85	0.28,29.00	0.91	0.409	2.49	0.29, 21.67
High school and above			1.00				1.00	
Residence								
Rural	0.01	0.989	1.01	0.45,2.24	-0.25	0.582	0.78	0.31, 1.92
Urban			1.00				1.00	
Marital status								
Unmarried (Single/Divorced/Widowed)	-0.34	0.133	0.71	0.46,1.11	-0.26	0.378	0.77	0.44, 1.37
Married			1.00				1.00	
Physical impairment								
No	0.71	0.020	2.03	1.12,3.67	0.79	0.039	2.20	1.04, 4.65
Yes			1.00				1.00	
Visual impairment								
No	0.50	0.027	1.65	1.06,2.58	0.61	0.030	1.84	1.06,3.19
Yes			1.00				1.00	
Duration of impairment (years)								
1–5	0.22	0.276	1.24	0.84,1.83	0.12	0.634	1.12	0.70,1.80
> 5			1.00				1.00	
Self-assessed memory								
Pool	-0.09	0.793	0.92	0.48,1.75	-0.63	0.128	0.54	0.24,1.20
Fair	0.08	0.816	1.08	0.55,2.13	0.26	0.525	1.30	0.58,2.94
Good			1.00				1.00	
Self-assessed hearing								
Pool	-0.78	0.047	0.46	0.22,0.99	-0.61	0.199	0.54	0.21,1.38
Fair	-0.14	0.728	0.87	0.39,1.94	0.51	0.305	1.66	0.63,4.37
Good			1.00				1.00	
Depression score								
1–10	0.24	0.580	1.28	0.54,3.01	1.34	0.034	3.82	1.11,13.22
11–20	0.61	0.182	1.84	0.75,4.51	1.36	0.036	3.91	1.09,13.99
21–30			1.00				1.00	
Myopia								
Pool	-0.39	0.444	0.68	0.25,1.83	-0.46	0.443	0.63	0.19,2.05
Fair	-1.00	0.040	0.37	0.14,0.96	-1.07	0.059	0.34	0.11,1.04
Good			1.00				1.00	
Farsightedness								
Pool	0.88	0.061	2.40	0.96,6.01	0.74	0.168	2.10	0.73,6.00
Fair	0.63	0.142	1.89	0.81,4.39	0.20	0.681	1.22	0.47,3.18
Good			1.00				1.00	
Complete tooth loss								
No	-0.07	0.740	0.93	0.60,1.44	-0.02	0.944	0.98	0.56,1.72
Yes			1.00				1.00	
Night sleep (h)								
≦ 7	-0.18	0.416	0.83	0.54,1.30	0.18	0.535	1.19	0.69,2.07
> 7			1.00				1.00	
Nap duration (min)								
≦ 30	0.46	0.019	1.59	1.08,2.34	0.72	0.003	2.05	1.28,3.28
> 30			1.00				1.00	
Social interactions								
No	-0.05	0.813	0.95	0.65,1.41	-0.07	0.771	0.93	0.58,1.49
Yes			1.00				1.00	
Smoking								
Never	-0.42	0.139	0.65	0.37,1.15	-0.39	0.268	0.68	0.34,1.35
Former smoker	-0.06	0.856	0.95	0.52,1.73	0.02	0.949	1.02	0.51,2.05
Current smoker			1.00				1.00	
Drinking								
Never	-0.03	0.921	0.98	0.59,1.61	-0.59	0.054	0.56	0.31,1.01
Former drinker	0.06	0.857	1.06	0.55,2.07	-0.22	0.568	0.80	0.37,1.72
Current drinker			1.00				1.00	
Retirement								
No	-0.79	0.134	0.46	0.16,1.28	-1.46	0.009	0.23	0.08,0.69
Yes			1.00				1.00	
Fuel use								
Clean fuels	0.34	0.208	1.40	0.83,2.36	0.40	0.212	1.48	0.80,2.76
Solid fuels			1.00				1.00	

## Discussion

Our study found that 37.8% of participants maintained stable cognitive function at a high level (H group), consistent with the findings of Zhang [29] and Mose [30]. However, the majority of hearing-impaired older adults in this study experienced cognitive decline, aligning with previous studies [29]. Specifically, 38.4% fell into the M group and 23.7% into the L group, with cognitive scores declining gradually (0–2 points) over the eight-year follow-up, which is in agreement with findings from Min [31] and Wu [32]. The average cognitive score (7.46 ± 5.16) was lower than that reported in Zhang’s study (13.63 ± 2.18)^29^, possibly due to differences in study populations. In the L group, some participants had scores close to 0, while Zhang’s lowest score was 4. This discrepancy may be attributed to Zhang’s inclusion of middle-aged and older adults without hearing impairments, whereas our study specifically focused on those with hearing impairment. It is important to note that previous studies have shown that cognitive changes in older adults with hearing loss may be influenced not only by hearing impairment itself but also by other common factors, such as aging, genetic factors, cardiovascular diseases, or chronic inflammation [33]. Our study further highlights the heterogeneity of cognitive function trajectories in this population, providing new evidence to inform the understanding of the relationship between hearing loss and cognitive decline.

It is worth noting that in CHARLS, hearing impairment was identified using a self-reported measure. Although this approach has inherent limitations, it offers practical feasibility for large-scale epidemiological surveys. Pure-tone audiometry remains the gold standard for assessing hearing loss; however, its implementation requires trained professionals, soundproof environments, and specialized equipment, which poses significant challenges in large cohort studies. As a result, self-reported hearing measures have been widely adopted in observational epidemiological research. For example, the English Longitudinal Study of Ageing (ELSA) and the U.S. Health and Retirement Study (HRS) have examined the association between hearing and cognition using self-reported data [12]. Similarly, the German LEILA75 + and AgeCoDe/AgeQualiDe cohorts reported consistent links between self-reported hearing and dementia [34]. Importantly, prior studies have demonstrated good concordance between self-reported and objectively measured hearing. In the ELSA cohort, overall consistency between the two methods was found to be moderate to high [35]. The Rotterdam Study reported that a single self-reported question on hearing difficulties achieved an AUC of 0.86 for detecting moderate or greater hearing loss [36], while a Finnish study found 100% sensitivity and 70.7% specificity in identifying moderate-to-severe impairment [37]. Moreover, a systematic review concluded that self-reported questions remain an effective alternative for hearing screening in older adults when objective assessments are not feasible [38].

In this study, it is also important to note that 87.53% of participants were permanent rural residents, most of whom had relatively low educational attainment: 39.84% had only a primary school education or less, and 47.48% were illiterate. These demographic characteristics are consistent with the typical profile of the older population in China. According to official CHARLS data, the overall response rate in the baseline survey was 80.5%, with a higher response rate in rural areas (94%) than in urban areas (69%). The lower response rate in urban areas is a common phenomenon in large-scale surveys conducted in developing countries [39]. Data from China’s Seventh National Population Census (2020) further confirm the rural concentration of older adults: 23.81% of the rural population was aged ≥ 60 years, significantly higher than in urban areas, and 45.83% of China’s total population aged ≥ 60 years lived in rural areass [40]. Moreover, a meta-analysis indicates that the prevalence of hearing loss among Chinese adults aged ≥ 60 years is approximately 69%, rising to 83.1% among those aged ≥ 75 years [41]. Together, these data support the representativeness of the rural distribution of hearing-impaired older adults in our study sample, reflecting broader national demographic characteristics.

This study further examined the factors influencing cognitive function trajectories in older adults with hearing impairment. The results indicated that age, education, physical and visual impairment, hearing status, farsightedness, depression, nap duration, and retirement status significantly affected cognitive changes. Consistent with previous studies [42], younger age was associated with better cognitive function, likely due to reduced oxidative stress and neuroinflammation.

The relationship between literacy levels, hearing impairment, and cognitive function warrants careful consideration [43]. In this study, participants with low literacy were significantly less likely than those with education beyond high school to belong to the H group, consistent with Su’s findings [44]. Low literacy levels may serve as a potential confounding factor in the accelerated cognitive decline associated with hearing impairment. According to cognitive load theory, individuals with lower literacy expend more cognitive resources on routine tasks [45], and hearing impairment further increases this burden, depleting resources available for higher-order functions such as memory and attention [18, 46]. Meanwhile, sensory deprivation theory [17] and social isolation theory [21, 47]both suggest that individuals with low literacy levels may inherently lack stimulation for language and speech centers as well as social interaction. Hearing impairment further exacerbates this lack of stimulation, thereby accelerating neurodegeneration and cognitive decline. Although the literacy levels observed in our sample reflect those of the general population, the possible influence of literacy on the mechanisms linking hearing loss to cognitive trajectories should not be overlooked. Future studies should further investigate these pathways to clarify the role of literacy in this relationship.

Our findings suggest a potential protective association between retirement and cognitive function in older adults with hearing impairment, which contrasts with much of the existing literature on cognitive engagement [48]. While our data indicate this relationship, it may reflect the unique characteristics of our study population, which consisted predominantly of individuals with limited education who may have held occupations offering minimal cognitive stimulation. However, several limitations must be acknowledged. Alternative explanations include selection bias, unmeasured confounding factors, or the possibility that retirement reduces stress rather than directly benefiting cognition in this population. Moreover, the generalizability of our findings to other populations may be limited. Future research incorporating detailed occupational information, longitudinal cognitive assessments, and diverse populations is warranted to confirm these observations and clarify the underlying mechanisms. Accordingly, we present this finding as a hypothesis-generating observation rather than a definitive conclusion.

Self-reported physical impairment was associated with greater cognitive decline, likely due to reduced mobility, lower physical activity, and decreased social participation, all of which contribute to cognitive deterioration [49]. Older adults without physical impairment were more likely to be in the M or H groups. The L group had a higher proportion of physically impaired individuals, with longer durations of impairment, confirming its role as a risk factor. Similarly, the absence of visual impairment was a protective factor, consistent with studies showing that vision decline significantly increases the risk of cognitive impairment and dementia [50]. Vision loss can limit reading, socializing, and other cognitively stimulating activities, leading to behavioral and cognitive decline [51, 52].

While both myopia and hyperopia are common vision issues, with research indicating that myopia may be a leading cause of visual impairment globally [53], their effects on cognitive function remain debated. Some studies suggest that uncorrected refractive errors contribute to cognitive declinee [54], whereas a large cohort study found no association between myopia and dementia [55]. In our study, we observed that myopia was a risk factor for cognitive decline, even though most participants were illiterate. One possible explanation is that our study population consisted of older adults with hearing impairment, for whom visual information may play a particularly important role in daily activities [56]. Myopia may reduce the ability to acquire distant or partial near visual information, such as difficulty recognizing people or reading road signs, which may indirectly limit outings, social engagement, and environmental exploration, thereby negatively affecting cognitive health [57, 58]. It is important to emphasize that this is a hypothetical inference, highlighting the need for further research on the distinct impacts of refractive errors on cognition, especially in populations with hearing impairments or low educational attainment.

Studies have shown that individuals with sensory impairments often have lower daily activity abilities and experience higher levels of depression and social isolation [8]. Reduced social interaction can contribute to cognitive decline in older adults [59]. In this study, hearing-impaired older adults with depression scores (score range: 0–30) of 1–10 points and 11–20 points were more likely to be classified in the H group. This suggests that individuals with no depression or mild depression are less likely to experience cognitive impairment. This finding aligns with Rong’s research [60], which indicated that hearing impairment is independently associated with poorer cognitive function and depressive outcomes.

In healthy younger individuals, napping has been shown to reduce drowsiness, enhance executive function, and promote memory consolidation, learning, and emotional processing, along with benefiting physical health [61]. However, Mantua’s research found that the effects of napping in older populations differ from those in younger ones [62]. While there is no direct evidence that napping is harmful, excessive napping has been linked to negative outcomes [43]. In this study, nap duration ≤ 30 min was associated with higher likelihoods of being classified in the M and H groups, suggesting that moderate napping (≤ 30 min) serves as a protective factor for cognitive function in hearing-impaired older adults. Conversely, longer daytime naps may interfere with nighttime sleep, leading to sleep deprivation, disruption of circadian rhythms, and poor sleep quality [63], which may ultimately impair cognitive function in older adults [64].

Additionally, this study found that older adults with poor hearing were less likely to be classified in the M group, reinforcing that poor hearing is a risk factor for cognitive decline. In a large-scale randomized controlled trial [65], hearing aid use did not demonstrate significant benefits for cognitive function. Similarly, another cohort study [66] reported no significant association between hearing aid use and overall cognitive changes. However, a systematic review and meta-analysis including 137,484 participants [67] found that hearing aids or other hearing correction devices were associated with a 19% reduction in the risk of long-term cognitive decline, and short-term cognitive tests showed an approximate 3% improvement. In a separate cohort study of 2,114 adults aged ≥ 50 years with self-reported hearing loss [68], participants with mild cognitive impairment who used hearing aids had a significantly reduced risk of all-cause dementia (HR: 0.73, 95% CI: 0.61–0.89). These findings highlight the need for future prospective studies with larger sample sizes and higher hearing aid usage rates to increase statistical power and further clarify the potential impact of hearing aids on cognitive function in older adults.

Notably, in this study, we included fuel use as a potential confounding factor to assess its impact on cognitive trajectories. Our findings, however, indicated no statistically significant association between fuel use and changes in cognitive function, which contrasts with previous studies [33].This result may suggest that, within our specific study population, the influence of fuel use on cognitive trajectories is limited, or it may be affected by measurement methods, variations in exposure levels, or other unmeasured factors. Nonetheless, this finding should be interpreted with caution, as the relationship between fuel use and cognitive health may vary across geographical regions, cultural contexts, and fuel types. Future research should consider more precise exposure assessment methods and explore the long-term effects of different fuel types and usage patterns to achieve a more comprehensive understanding of this potential association.

Using GBTM, this study clearly examined the trajectories of cognitive function changes in hearing-impaired older adults over time, identifying key risk factors: advanced age, physical and visual impairment, high depression scores, long naps, illiteracy, poor hearing, farsightedness, and non-retirement. Therefore, early cognitive screening and timely interventions are recommended. Encouraging older adults with hearing impairment to enhance hearing, engage in social activities, address depressive symptoms, and avoid prolonged napping may help maintain cognitive health.

Despite its contributions to the existing literature, this study has several limitations. First, hearing status was self-reported by participants, which may introduce reporting bias. Second, due to substantial missing data, not all known factors affecting cognitive function were included in our analysis. In addition, we cannot rule out the possibility of reverse causation, as early cognitive decline may influence self-reported hearing status. Other potential confounders, such as genetic factors or urban pollution, might also affect cognitive trajectories but were not fully accounted for in the CHARLS survey. Finally, the study population was limited to China, therefore future research can include larger, multi-country samples to enhance the generalizability of the findings.

## Conclusions

The trajectories of cognitive function changes in Chinese older adults with self-reported hearing impairment can be categorized into three groups: the Low-functioning decline group, the Middle-functioning decline group, and the High-functioning stabilization group. Factors influencing these trajectory groups include age, education level, physical impairment, visual impairment, hearing status, farsightedness, depression, nap duration, and retirement status. Given the limited generalizability of these preliminary findings, further studies are warranted to examine the potential confounding and mediating relationships among these factors before they can meaningfully inform early intervention strategies and policy development.

## Data Availability

The data used in this study were obtained from the China Health and Retirement Longitudinal Study (CHARLS), a publicly available dataset administered by Peking University. Access to the data requires registration and approval through the CHARLS website: http://charls.pku.edu.cn.
